# The Impact of Stress Hormones on Post-traumatic Stress Disorders Symptoms and Memory in Cardiac Surgery Patients

**DOI:** 10.5681/jcvtr.2014.018

**Published:** 2014-06-30

**Authors:** Jahan Porhomayon, Sergei Kolesnikov, Nader D Nader

**Affiliations:** University at Buffalo, Buffalo, New York, USA

**Keywords:** Cardiac Surgery, Chronic Post-Traumatic Stress Disorder, Adrenal Cortex Hormones

## Abstract

The relationship and interactions between stress hormones and post-traumatic stress disorder (PTSD) are well established from both animal and human research studies. This interaction is especially important in the post-operative phase of cardiac surgery where the development of PTSD symptoms will result in increased morbidity and mortality and prolong length of stay for critically ill cardiac surgery patients. Cardiopulmonary bypass itself will independently result in massive inflammation response and release of stress hormones in the perioperative period. Glucocorticoid may reduce this response and result in reduction of PTSD symptom clusters and therefore improve health outcome. In this review, we plan to conduct a systemic review and analysis of the literatures on this topic.

## Introduction

The definition of PTSD, like most other psychiatric disorders, relies on descriptive methodology.^1^ In the DSM-5, the diagnosis of PTSD has undergone multiple, albeit minor, changes. These changes include shifting PTSD placement from within the anxiety disorders into a new category of traumatic and stressor-related disorders, alterations in the definition of a traumatic event, shifting of the symptom cluster structure from three to four clusters, the addition of new symptoms including persistent negative beliefs and expectations about oneself or the world, persistent distorted blame of self or others, persistent negative trauma-related emotions, and risky or reckless behaviors, and the addition of a dissociative specific.^2^ PTSD is a known complication of critical illness.^3^ The development of PTSD and agitation in critically ill patient is multifactorial. Sedation strategy in the intensive care unit is one factor related to the development of mental health disorders.^4 ,5^ PTSD has also been reported in patients after cardiac surgery and intensive care unit survivors.^6,7^ Recalls have been reported with memories such as nightmares, anxiety and pain with little or no recall of events.^8^ A high number of these traumatic memories after cardiac surgeries have been shown to be a significant risk factor for the later development of PTSD. One important factor impacting the incidence of PTSD in cardiac surgery patients is related to the use of stress hormones.^9^ The numbers of traumatic memories are altered with the administration of catecholamines and cortisol.^3,10,11^ Previous research had demonstrated that the higher doses of cortisol results in better memory consolidation. Conversely, the prolonged administration of beta-adrenergic antagonists during the recovery phase after cardiac surgery resulted in a lower number of traumatic memories and a lower incidence of stress symptoms at 6 months after surgery.^3,12,13^ In order to better understand the role of stress hormones in PTSD, we conducted this systemic review.

## Prevalence of PTSD in Cardiac Surgery

The prevalence of PTSD in cardiac surgery ranges from 5% to 14.7% based on different database.^14,15^ The study by Dao et al^16^ performed a retrospective analysis of the 2006 Nationwide Inpatient Sample database. The Nationwide Inpatient Sample database provides information on approximately 8 million US inpatient stays from about 1000 hospitals. He evaluated potential confounding group, demographic and medical variables. Hierarchic logistic regression was used with forced order entry of depression, posttraumatic stress disorder, and comorbid depression and posttraumatic stress disorder recorded. He reported that deceased patients were more likely to have had PTSD (alive, 13.4%; deceased, 56.1%; P< 0.001), and co-morbid depression and PTSD (alive, 7.8%; deceased, 48.5%; P< 0.001). After adjusting for potential confounding factors, he concluded that PTSD (odds ratio, 2.09; 95% confidence interval, 1.65-2.64), and comorbid depression and posttraumatic stress disorder (odds ratio, 4.66; 95% confidence interval, 3.46-6.26) had an increased likelihood of in-hospital mortality compared with those still alive. This is in comparison with the study by Tully et al^17^ who reported less than 5% of cardiac surgery patients met current criteria for PTSD before cardiac surgery after diagnostic interview.^6^

## Pathophysiology

Critically ill cardiac surgery patients are at risk for PTSD and memory impairment or recalling traumatic memory events.^18^ These memory events are important in developments of PTSD.^19^ Previous research has shown a clear association between these memories and PTSD. There is clear connection between these memories and interaction between the noradrenergic, the glucocorticoid^20-22^ and the endocannabinoid system. Critically ill surgical patients are generally treated with sedative and adrenergic or glucocorticoid drugs^23^; among them propofol is known to impact endocannabinoid signaling.^24^ The endocannabinoid system is an important regulator of HPA-axis activity during stress^25^, an effect which has also been demonstrated in humans.^26^ High dose corticosteroids have been reported to reduce symptoms of acute stress and PTSD in poly-trauma patients and in animal studies.^27-29^ The underlying mechanism of action remains largely unclear. Evidence from clinical and animal studies suggests that there is a “window of opportunity” in the early aftermath of trauma to help those who are vulnerable to the development of chronic PTSD.^30,31^ Several researchers have demonstrated that the administration of cortisol to critically ill surgical patient results in a significant reduction of PTSD symptoms after recovery without influencing the number of traumatic memories.^32,33^ Nevertheless, the manipulation of glucocorticoid endocannabinoid interaction during traumatic memory consolidation and the use of steroids for prophylaxis and treatment of PTSD should be tested in larger controlled studies.^34,35^ Hauer et al^36^ examined the relationship between serum cortisol, traumatic memories and PTSD in survivors of patients from a mix ICU after acute respiratory distress syndrome (ARDS). He noticed that during evaluation, patients with multiple traumatic memories had significantly lower basal serum cortisol levels when compared to patients with no or only 1 category of traumatic memory, with no differences in peak cortisol levels after corticotropin stimulation between both subgroups. There was a significant negative correlation between basal cortisol levels and the number of traumatic memories present. PTSD symptom scores correlated with the number of traumatic memories but not with cortisol levels. These findings indicated that lower baseline cortisol levels in long-term survivors of ARDS are associated with an increased incidence of traumatic memories from the ICU, and that more traumatic memories are related to a higher incidence and intensity of PTSD symptom.

## Methods

**Figure 1:**
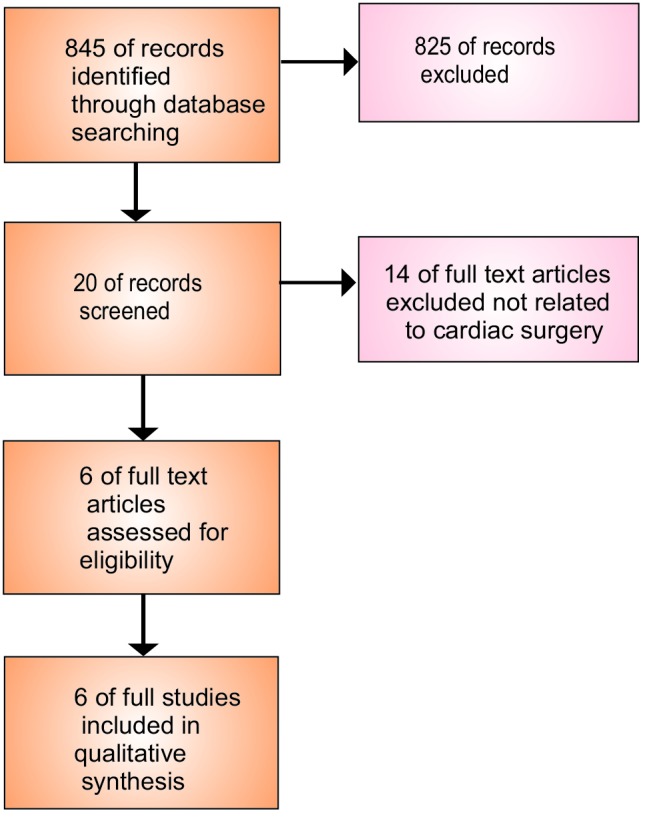
The flow diagram for the systematic review.

We searched the electronic databases Medline (from 1966 to March 2013) and Embase (from 1980 to March 2013), as well as the Cochrane Library using the query terms “Intensive Care Unit,” and “Mental Health,” yielded 845 articles. Narrowing the search by adding additional queries such as, “post-traumatic stress disorders” “cardiac surgery,” and “stress hormones” yielded 20 articles. 5 prospective randomized controlled trials (RCTs) and 1 observational study were identified for review. Furthermore, we reviewed reference lists of the original and reviewed articles to search for more studies. Only those that were published as full-length articles were considered. No language restriction was applied (Figure 1). Two studies by Schelling et al^37,38^ were in mix ICUs. However, after reviewing the articles, we included them in final analysis since they involved cardiac patients.

## Current Studies Assessing the Role of Stress Hormones in PTSD

Several retrospective and prospective clinical studies have evaluated the role of stress hormones in PTSD. The study by Schelling and colleagues was a retrospective case-controlled analysis in a 20 bed mix ICU including cardiac patients. 27 patients served as controls who received standard therapy for septic shock. These patients were compared with an equal number of patients who received hydrocortisone in addition to standard treatment. PTSD was diagnosed with the Posttraumatic Stress Syndrome-10 inventory, a self-report scale for diagnosis of PTSD. Health-related quality of life was measured using the Medical Outcomes Study Short-Form Survey which consists of 36 questions. Patients who received hydrocortisone during septic shock had a significantly lower incidence of PTSD than patients who received standard treatment only (5 of 27 vs. 16 of 27; P= 0.01) and had significantly higher scores on the mental health index of the Medical Outcomes Study Short-Form health-related quality-of-life questionnaire (68 vs. 44 points; P= 0.009). The authors concluded that administration of stress doses of hydrocortisone in doses equivalent to the maximal endocrine secretion rate during septic shock reduces the incidence of PTSD and improves emotional well-being in survivors.^37^ The same authors conducted a randomized double blind study assessing the impact of steroids on PTSD in critically ill patients with septic shock in a mix ICU including cardiac patients. 20 patients were recruited. Eleven patients had received placebo and nine stress doses of hydrocortisone. PTSD was diagnosed 31 months after ICU discharge using DSM-IV criteria. Only one of nine patients from the hydrocortisone group developed PTSD, compared with seven of 11 patients in the placebo group (P= 0.02). There was no significant difference with regard to the number of categories of traumatic memory between the hydrocortisone and placebo groups. The authors concluded that the administration of hydrocortisone during septic shock in a dosage similar to the endogenous maximal production rate was associated with a lower incidence of PTSD in long-term survivors.^38^ Schelling et al also conducted a randomized control study on 91 patients to receive standard dose hydrocortisone therapy or standard treatment during the perioperative period. Of 48 available patients at 6 months after cardiac surgery, 26 had received stress doses of hydrocortisone and 22 standard treatments. Traumatic memories and PTSD symptoms were diagnosed with previously validated questionnaires. As compared with patients after standard therapy, patients from the hydrocortisone group had significantly lower chronic stress symptom scores (P<0.05). There was no significant difference regarding the number or type of traumatic memories between the hydrocortisone and the standard treatment groups. Therefore, stress doses of hydrocortisone in patients undergoing cardiac surgery was associated with a lower intensity of chronic stress and PTSD symptoms.^15^ Weis et al evaluated immunologic effects (especially IL-6 to IL-10 ratio) of stress doses of hydrocortisone in a high-risk group of patients (n=32) after cardiac surgery with cardiopulmonary bypass in a Prospective, randomized, double-blinded, placebo-controlled trial. The patients from the hydrocortisone group (n= 19) had significantly lower levels of IL-6 and higher levels of IL-10, resulting in an attenuated change in IL-6/IL-10 ratio [28.7 (6.4/128.7) vs. 292.8 (6.5/534.6) 4 hours after cardiopulmonary bypass; P< 0.001]. Patients in the hydrocortisone group had a shorter duration of catecholamine support [1 (1/2) vs. 4 (2/4.5) days; P= 0.02], a shorter length of stay in the intensive care unit [2 (2/3) vs. 6 (4/8) days; P= 0.001], and a lower incidence of postoperative atrial fibrillation (26% vs. 59%; P= 0.04). He concluded that stress doses of hydrocortisone attenuate the evolution of IL-6/IL-10 ratio in patients with systemic inflammatory response syndrome after cardiac surgery, which seems to be associated with an improved outcome.^39^

Tarsitani et al investigated the perioperative factors associated with the development of PTSD in patients who underwent cardiac surgery. He designed a prospective observational study and recruited 128 consecutive patients scheduled for elective cardiac surgery with cardiopulmonary bypass. Six months after surgery, participants were mailed the modified version of the PTSD Inventory 10. Of the 71 patients who completed the questionnaire and mailed it back at follow-up, 14 (19.7%) received a diagnosis of PTSD. Seven of 13 female patients who were not treated with beta-blockers received a diagnosis of PTSD compared with 0 of 12 who were treated with beta-blockers (P= 0.005). In a general linear model, including sex and beta-blocker treatment as predictors, the PTSD Inventory 10 score was significantly predicted by beta-blockade (F= 4.74, P= 0.033), with a significant interaction between sex and beta-blockade (F= 9.72, P= 0.003). He concluded that the use of beta-blockers might be protective against the development of PTSD in women after cardiac surgery.^40^ Krauseneck et al also demonstrated in prospective observational study that beta-Adrenergic stimulation with epinephrine enhances memory for adverse experiences in males but not in females whereas beta-blockade selectively reduces memory for post-operative adverse events and PTSD symptoms in females^13^ (Table 1).

**Table 1:** Studies Evaluating the Impact of stress Hormones on the Development of PTSD in Cardiac Surgery Population

**Author**	**Design**	**Number**	**Setting**	**Intervention**	**P value**
Schelling et al^37^	Retrospective	27	Mix ICU	Hydrocortisone vs control	0.01
Schelling et al^38^	Prospective	20	Mix ICU	Hydrocortisone vs control	0.02
Schelling et al^23^	Prospective	91	Cardiac ICU	Hydrocortisone vs control	<0.05
Weis et al^39^	Prospective	19	Cardiac ICU	Hydrocortisone vs control	<0.001
Tarsitani et al^40^	Prospective	128	Cardiac ICU	Beta blocker vs control	<0.033
Krauseneck et al^18^	Prospective	128	Cardiac ICU	Beta blocker vs control	<0.02

## Discussion

Cardiac surgery and trauma induce severe inflammation and stress response. There is also dysfunction of brain structures, particularly the amygdala, locus coeruleus, hippocampus, nor-adrenergic system and hypothalamic-pituitary-adrenal axis (HPA) during stressful events such as trauma and cardiac surgery.^41,42^ The activations of neurotransmitter and neurobiological systems during such a events, may account for the primary symptoms of PTSD. Severe psychological trauma and stress^43^ results in the parallel activation of these systems, and is necessary for survival. The interaction of stress and the HPA axis, leading to the release of glucocorticoids in humans by the adrenal cortex is well documented.^44^ It is also established that in humans, steroids can influence memory processes by acting on brain structures.^21^ Stress and heightened cortisol concentrations seem to enhance memory.^33^ Emerging evidence indicate that the use of hydrocortisone may not only decrease the incidence of PTSD in cardiac surgery but also reduce systemic inflammation response during or after cardiopulmonary bypass in coronary bypass surgery.^42,45^

The decreased levels of cortisol, the increased responsiveness of glucocorticoid receptors, and the increased sensitivity of the HPA negative feedback inhibition are associated with the occurrence of PTSD.^46^ In order to further assess the interaction between stress hormones and PTSD, Yehuda and colleagues measured dopamine, norepinephrine, and epinephrine concentrations in 22 male patients with PTSD (14 inpatients and eight outpatients) and in 16 non-psychiatric normal males. The PTSD inpatients showed significantly higher excretion of all three catecholamines compared with both outpatients with PTSD and normal controls. Dopamine and norepinephrine, but not epinephrine, levels significantly correlated with severity of PTSD symptoms in the PTSD group as a whole. In particular, these catecholamines seemed related to intrusive symptoms. The findings support the theory of greater sympathetic nervous system activation in PTSD, and suggest that increased sympathetic arousal may be closely linked to severity of certain PTSD symptom.^47^

Low levels of cortisol leads to a higher incidence of PTSD, months after critical illness resolution.^48^ There is a close link between HPA axis, cortisol and PTSD symptoms. Our knowledge in regard to treatment of PTSD and its interaction with stress hormones is evolving. Additional research and multi-center trials are needed to better define and delineate this relationship.

## Conclusion

Critically ill cardiac surgical patients are at increased risk of PTSD due to massive stress response from cardiopulmonary bypass. Manipulation of the stressful hormonal response can result in reduction of traumatic memory and PTSD development. This could also result in new approaches for prophylaxis and treatment of stress related disorders.

## Ethical issues

Not applicable.

## Competing interests

Authors declare no conflict of interest in this study.
